# A cluster randomized controlled trial to increase the availability and acceptability of voluntary medical male circumcision in Zambia: The Spear and Shield Project

**DOI:** 10.1016/S2352-3018(15)00042-9

**Published:** 2015-05-01

**Authors:** Stephen M Weiss, Robert Zulu, Deborah L Jones, Colleen A Redding, Ryan Cook, Ndashi Chitalu

**Affiliations:** a Department of Psychiatry & Behavioral Sciences, University of Miami Miller School of Medicine, Miami, Florida, USA; b Department of Medicine, University of Zambia School of Medicine, Lusaka, Zambia; c Department of Psychology, Cancer Prevention Research Center, University of Rhode Island, Kingston, Rhode Island, USA

## Abstract

**Background:**

Widespread voluntary medical male circumcision (VMMC) in Africa could avert an estimated 3·436 million HIV infections and 300,000 deaths over the next 10 years. Most Zambian men, however, have expressed little interest in undergoing VMMC. This study tested the effect of an intervention designed to increase demand for VMMC among these “hard to reach” men.

**Methods:**

This cluster randomized controlled trial was conducted from 2012 to 2014 in Lusaka, Zambia (HIV prevalence = 20·8%). 13 Community Health Centers (CHCs) were stratified by HIV voluntary counseling and testing (VCT) rates and patient census and randomly assigned (5:5:3) to Experimental, Control or Observation Only conditions. CHC health care providers at all 13 sites received VMMC training. Trial statisticians did not participate in randomization. 800 uncircumcised HIV-, post-VCT men, 400 per condition, were recruited; female partners were invited to participate. The primary outcome was the likelihood of VMMC by 12 months post-intervention. The trial registration is NCT 01688167.

**Findings:**

161 participants in the Experimental condition underwent VMMC as compared to 96 Control participants [adjusted odds ratio = 2·45, 95% CI = (1·24, 4·90) *p* = ·0166]. Post-VMMC condom use among Experimental condition participants increased compared to baseline, with no change among Control participants. No adverse events related to study participation were reported.

**Interpretation:**

The Spear and Shield intervention combined with VMMC training was associated with a significant increase in the number of VMMCs performed as well as in condom use among “hard to reach” Zambian men. Results support the importance of comprehensive HIV prevention programs that increase supply of and demand for VMMC services.

**Funding:**

NIH/NIMH R01MH095539.

## INTRODUCTION

Evidence of the effectiveness of voluntary medical male circumcision (VMMC) in decreasing the risk of HIV infection for men in Southern and Eastern Africa by 51–60% was so compelling that three randomized clinical trials were halted prematurely due to positive preliminary results. ^1,2^ Follow-up studies of up to 5·5 years post-circumcision have reported increased levels of protection (up to 67–73%) confirming the long term protective effect of VMMC. ^3–5^ These findings support concurrent observations that behavioral disinhibition (e.g., reduction in condom use, increase in multiple partners) did *not* occur in newly circumcised men. ^6,7^ Mathematical modeling projections suggest that widespread VMMC in Africa could avert up to 3·4 million HIV infections and 300,000 deaths over the next 10 years and an additional 3·7 million infections and 2·7 million deaths in the following 10 years. ^8^

With a population of nearly 13 million, Zambia has high HIV prevalence (19.7% urban) and incidence (4% urban) among those 15–49 and a low rate of male circumcision (MC; 12%). ^9^ The WHO/GPA public health recommendations were initially codified by the Zambian Ministry of Health into a five year plan, the Zambian National Male Circumcision Strategy and Implementation Plan 2010–2015, with the goal of performing 1·9 million male circumcisions (80% of the eligible male population) by 2015, or 400,000 circumcisions per year. The Plan has since been extended until 2020, with a modified goal of 1·25 million MCs by 2015.

The initial enthusiasm for VMMC led to long waiting lines of prospective patients at hospitals and community health centers, and as of October, 2014, a total of over 700,000 circumcisions had been reported by the GRZ Ministry of Community Development and Maternal Health. However, this represents ~37% of the national goal and the Zambia Sexual Behaviour Survey ^9^ and subsequent studies ^10^ forecast a less optimistic portrait of VMMC acceptability and uptake among the remaining population of uncircumcised Zambian men. While studies conducted in several sub-Saharan African countries have found at least 65% of the men surveyed expressing willingness to be circumcised, ^10^ the Zambia survey published in 2010 indicated that *over 80% of uncircumcised men interviewed had no interest in undergoing VMMC.* Among those surveyed, major impediments to undergoing VMMC as a risk reduction strategy included fear of pain, concerns about post-surgical sexual performance and satisfaction, cultural tradition and partner preferences. These perceptions suggest the need for a more comprehensive strategy for increasing acceptability (demand) as well as availability (supply) of medical circumcision services in Zambia.

To optimize the potential benefits of VMMC, innovative community-level interventions are needed to convince “hard to reach” -uncircumcised Zambian men, i.e., men who express no interest in undergoing VMMC, that VMMC is a viable means of reducing their risk of HIV infection. This cluster randomized controlled trial sought to determine the impact of increasing both the availability and the acceptability of VMMC among high risk men with little interest in circumcision, using a comprehensive sexual risk reduction/VMMC promotion intervention designed to systematically scale up both supply of and demand for VMMC services. As health care providers at all 13 study sites received VMMC training prior to study onset, changes in the likelihood of VMMC would be due to the presence (or absence) of the Experimental or Control conditions in comparison with the Observation only condition. It was hypothesized that the Experimental intervention would significantly increase the likelihood of VMMC with no increase in sexual risk behaviors over the 12 month follow-up period as compared to the Control condition.

## METHODS

### Study Design

The trial was undertaken and reported in accordance with CONSORT guidelines for cluster-randomized trials ^11^ and is registered on Clinicaltrials.gov, number NCT 01688167. This cluster randomized controlled trial was conducted at 13 urban community health centers (CHCs) in Lusaka District, Zambia. A cluster-randomized design was chosen in order to avoid cross-contamination between conditions within clinics. CHCs were randomized to three conditions, Experimental, Control, Observation-only, in a 5-5-3 ratio, respectively. The Observation only arm was designed to control for the effect of media, public health campaigns and other secular influences, as well as for the effect of having circumcision services becoming newly available in the CHC catchment area. Clinics were identified in consultation with the Lusaka Provincial Health Office and were selected based on: 1) > 50 HIV voluntary counseling and testing (VCT) participants per month, 2) no trained CHC personnel currently performing circumcisions on a regular basis, 3) at least 3 health care providers available at each site for circumcision training, and 4) a minimum of 2 VCT counselors (or equivalent) available at each site for sexual risk reduction training.

### Participant Recruitment and Enrollment

Male participants were recruited between January 2012 and September 2013 from 10 CHCs (n = 5 Experimental, n = 5 Control) following VCT; participant follow-up was completed by November 2014. Eligible participants were at least 18 years of age, HIV-negative, uncircumcised, and had not proactively requested or planned for VMMC at the time of enrollment. Men seeking circumcision services and those with genital abnormalities requiring MC, having diseases of the foreskin, or congenital or acquired penile abnormalities that required repair were excluded. Men were encouraged, but not required, to enroll with their female sexual partner. All study participants were compensated K20 Zambian Kwacha (~US$4) for each assessment, and were followed for one year, with provision for an additional 3 month post-circumcision assessment for those who underwent VMMC during the course of the study. Assessors reminded all study participants to complete a post-circumcision assessment after they completed the post-intervention assessment, if they underwent circumcision. Prior to the onset of study procedures, approval was obtained from the University of Miami Institutional Review Board, the University of Zambia Research Ethics Committee and from the Lusaka District and Provincial Health Offices. Prior to study initiation, approval to initiate the study at each clinic was obtained from clinic, District, and Provincial leadership. All participants provided written informed consent in English, Nyanja or Bemba prior to study enrollment.

### Sample size determination, randomization and masking

It was initially determined that the study would achieve 80% power to detect a significant difference between conditions with 12 clinics allocated in a 1:1 ratio (Experimental, Control) with each clinic recruiting 8 cohorts of 10 men for a total of 960 men. This analysis was based on VMMC rates of 5–10% in the Control arm, rates of 30–40% in the Experimental arm, and intracluster correlation coefficients (ICC) up to 0.27 with a two tailed test at the 0·05 level. ^12^ From these two estimated VMMC rates, the maximum “allowable” ICC was determined by examining multiple power curves in which the effect and sample sizes were fixed and power was plotted as a function of ICC. The range of plausible VMMC rates in the Control arm was based on the VMMC prevalence in the study location, and plausible rates in the Experimental arm were based on previous biobehavioral research by the study team (while maintaining an effect size that would be clinically significant). In addition, the upper bound of the number of clusters was derived from the number of clinics that met inclusion criteria and were available to implement and carry out the intervention in the study location and time period. However, due to logistic and staffing limitations, the total number of experimental and control clinics was reduced to 10. The study power was re-assessed and it was determined that adequate power would be maintained with 10 clinics and 800 male participants.

Randomization was conducted using a random allocation computer generated sequence undertaken by Zambian investigators; trial statisticians did not participate in randomization. Prior to randomization, clinics were stratified by patient census and monthly VCT rates to ensure equal numbers of large-, medium- and small-census CHCs in each condition. Based on the determination of the 3 largest, three smallest and seven mid-sized CHCs, a staged randomization procedure was conducted, as follows: Stage 1: the three largest CHCs were randomly assigned by computer, with random ordering of assignment, i.e., random ordering of the Experimental, Control and Observation Only conditions; Stage 2: the smallest 3 CHCs were allocated to condition in a similar manner; Stage 3: the 7 mid-sized CHCs were randomized in two “sub-stages” A and B. Substage A randomized three randomly selected mid-sized CHCs to the 3 conditions. Sub-stage B randomly assigned the remaining 4 CHCs to either Experimental or Control conditions. In this way, all CHCs had an equal chance of being chosen for any of the three conditions until their quota was filled, i.e., the Observation Only condition included only 3 CHCs. Clinics were notified of their condition assignment using premade sealed envelopes. Within clinics, post VCT, all interested men were screened for study eligibility, in accordance with the criteria noted previously.

### Procedures

All 13 CHC sites received surgical training to perform VMMC three months prior to randomization. Three providers from each of the 13 sites participated in a two week circumcision training program in accordance with the VMMC guidelines from the Health Professions Council of Zambia. ^13^ Training followed the WHO VMMC training manual and focused on the Dorsal Slit method, which is recommended by the Surgical Society of Zambia and the Zambia Ministry of Health. Following randomization, one month prior to the onset of recruitment, two VCT counselors or nurses from the 5 experimental sites were trained to conduct the sexual risk reduction/MC promotion intervention (“Spear and Shield”), and two VCT counselors or nurses at the 5 control sites were trained to provide the time-matched endemic disease prevention program. Trainees from each of the Experimental sites participated in a 2-day intensive training workshop on the intervention and study protocol, after which each trainee conducted 2, 4-session groups at their CHC supervised by a staff trainer from the University of Zambia School of Medicine. Emphasis was placed equally on training techniques (“training the trainer”) as well as intervention content to provide training skills to the newly trained CHC staff, to prepare them to train others within their CHC to conduct the Spear and Shield intervention as well as becoming potential trainers for staff from neighboring CHCs. At Observation Only sites, only monthly data on VCT and VMMCs were collected.

The Spear and Shield intervention and assessments were in local languages and culturally tailored from qualitative data from 3 focus groups and 12 key informant interviews on male and female attitudes, preferences and beliefs related to VMMC. The intervention and assessment materials were guided by the Information, Motivation, Behavior Model (IMB). ^14^ The comprehensive risk reduction intervention consisted of four weekly 90-minute manualized group sessions. Female partners of participants were invited to participate in a 4-session program comparable to that of their male partners, i.e., depending on whether their partner was in the Experimental or Control condition. Site interventionists were evaluated using intervention checklists, audio tapes of sessions, and ongoing monthly supervision for quality assurance. To further ensure fidelity, 10% of all sessions were reviewed by the US research team. A refresher training session was held after one year with all Experimental sites.

### Spear and Shield intervention

Session One addressed HIV/STDs, safer sex, male condoms, male circumcision and sexual communication. The session reviewed the protection offered by male condoms, followed by “hands-on” demonstrations, including practice with penis models. Multiple strategies for protecting against HIV were discussed, emphasizing VMMC. The session addressed VMMC as a permanent method of risk reduction, and provided a forum for discussion of the VMMC procedure, concerns, limitations and beliefs. Cognitive behavioral (CB) skill training heightened participants’ awareness of reactions to VMMC, reframing thoughts that impede VMMC uptake, condom use, and sexual communication. Participants received a week’s supply of male and/or female condoms after sessions 1–3.

Session Two reviewed topics from the first session. Female condoms were introduced and correct usage was demonstrated using female anatomical charts. CB skills were applied to sexual negotiation and improving communication techniques in relationships. The potential benefits of VMMC for female partners were addressed.

Session Three reviewed previous sessions and introduced novel products (e.g., PrEP, microbicides) to reinforce the concept of multiple protective strategies. Participants shared experiences, concerns and attitudes regarding female condom, and perceptions of partners’ reactions to VMMC. Role plays stimulated participants’ problem solving strategies, applying CB skills to safer sex, sexual communication and VMMC. A peer who had undergone VMMC shared his experiences with the group, followed by Q & A.

Session Four reviewed methods of HIV risk reduction. Participants created individual risk reduction plans and discussed options for protection. The session included a presentation on the VMMC procedure by a CHC VMMC provider, who discussed benefits and risks, post-VMMC recovery and resumption of sexual activities. Avoidance of high risk sexual behavior under the influence of alcohol or drugs was discussed, and CB skills were used to address conflict resolution, communication and sexual negotiation. Participants received a month’s supply of condoms.

Control condition. Participants in the Control condition sites attended 4 video-based time-matched “attention-control” group sessions on endemic disease prevention strategies (e.g., TB, malaria, cholera, waterborne diseases) and received male and female condoms equivalent to the Experimental condition.

### Outcomes

Outcomes addressed in this manuscript are VMMC and sexual risk behavior. Throughout the course of the study, VMMCs were verified by study staff. Verification was completed through clinic record reviews (50%), VMMC provider interviews (24%), or voluntary physical examinations (26%). Fifteen participants who reported that they had undergone VMMC but were unable to be verified were excluded from the primary analyses, but were included in a sensitivity analysis.

Primary study outcomes were measured at the individual level, and all results are presented at the individual level. All assessments (baseline, post intervention, 6 and 12 months post-intervention, 3 months post VMMC) were completed using an Audio Computer-Assisted Self-Interview (ACASI). For the purposes of this paper, Experimental vs Control odds of VMMC, post-VMMC behavioral disinhibition, and Experimental/Control/Observation site VMMC comparisons (excluding study participants) over the course of the study were the outcomes of interest. Other key issues, e.g., women’s role in male VMMC decision-making, post-VMMC sexual functioning/satisfaction, Stages of Change comparisons, were beyond the scope of this paper and will be addressed in subsequent publications.

The conceptual model for categorization of behavior change was the Transtheoretical Model, ^15^ which utilized stages of change, among other variables, to categorize participant’s VMMC intentions, behaviors and shifts in readiness over the course of the study to consider VMMC as an acceptable HIV prevention method. The stages were Pre-Contemplation (has never thought of becoming circumcised prior to study participation or not considering undergoing circumcision within the next six months); Contemplation (has considered the possibility of becoming circumcised sometime in the next six months); Preparation (making plans to undergo circumcision within the next thirty days); Action (undergoing VMMC) and Maintenance (abstaining from sexual activity for six weeks until completely healed from the surgery and maintaining pre-surgical sexual risk reducing strategies e.g., condom use, limiting number of sexual partners, always using condoms when drinking or using drugs). At baseline, most (88%) study participants reported being either Pre-Contemplators or Contemplators; 12% of participants were in the Preparation stage. Parenthetically, we conducted our principal analysis both including and excluding this 12%, finding minimal difference in Experimental vs Control study outcomes in either case.

### Statistical analysis

Preliminary analyses included descriptions of demographic characteristics and bivariate tests of association between randomized condition and demographics and VMMC status and demographics. In order to describe the pattern of incidence of VMMCs over time, Kaplan-Meier curves were generated.

All primary and secondary analyses included random intercepts in order to account for the treatment of participants within cohorts and the cluster-randomized design. Prior to the primary analysis, intraclass correlations were computed using a “null model” including only intercept terms. The primary analysis was a multilevel logistic regression comparing the odds of VMMC between conditions, adjusting for variables 1) chosen *a priori* as potential confounders (i.e., age, education level, and baseline stage of readiness for VMMC) and 2) significantly associated with VMMC in bivariate analyses and demonstrating evidence of imbalance between randomized conditions. Results of the primary analysis are reported as adjusted odds and odds ratios. In order to evaluate the overall performance of the model, two R^2^ statistics were calculated using a “marginal” R^2^, indicating the “variance explained” by the fixed effects portion of the model, and a “conditional” R^2^, indicating the total variance explained by the fixed and random portions of the model.

In addition to the primary analysis, an unadjusted comparison of the Experimental vs. Control condition was made, and two sensitivity analyses were completed using the same methodology as the primary analysis. The first included 15 participants who self-reported undergoing VMMC but were unable to be verified, and the second excluded 99 participants who inadvertently entered the study already planning to undergo VMMC. As a secondary analysis, a linear mixed model was used to examine condom use over time for those participants who underwent VMMC, accounting for the varying lengths of time to VMMC. This analysis treated condom use as a continuous outcome and included randomized condition, time, and the interaction between condition and time as predictors, with the same random effects as the primary analysis.

Finally, monthly clinic-wide VMMC and HIV testing data was summed over 36 months of study activity to examine proportions of individuals undergoing VMMC (excluding study participants) from all 13 CHCs. The numerator of each proportion was the total number of non-study VMMCs done at the clinics and the denominator was the total number of men testing negative for HIV (i.e., those eligible for VMMC). Using these proportions, odds and odds ratios were calculated in order to compare non-study VMMCs between conditions (i.e., Experimental, Control, and Observation-only).

### Role of the funding source

The sponsor of this study had no role in the study design, data collection, data analysis, data interpretations, or writing of the report. The corresponding author had full access to all of the data in the study and had final responsibility for the decision to submit this manuscript.

## RESULTS

**Figure 1:**
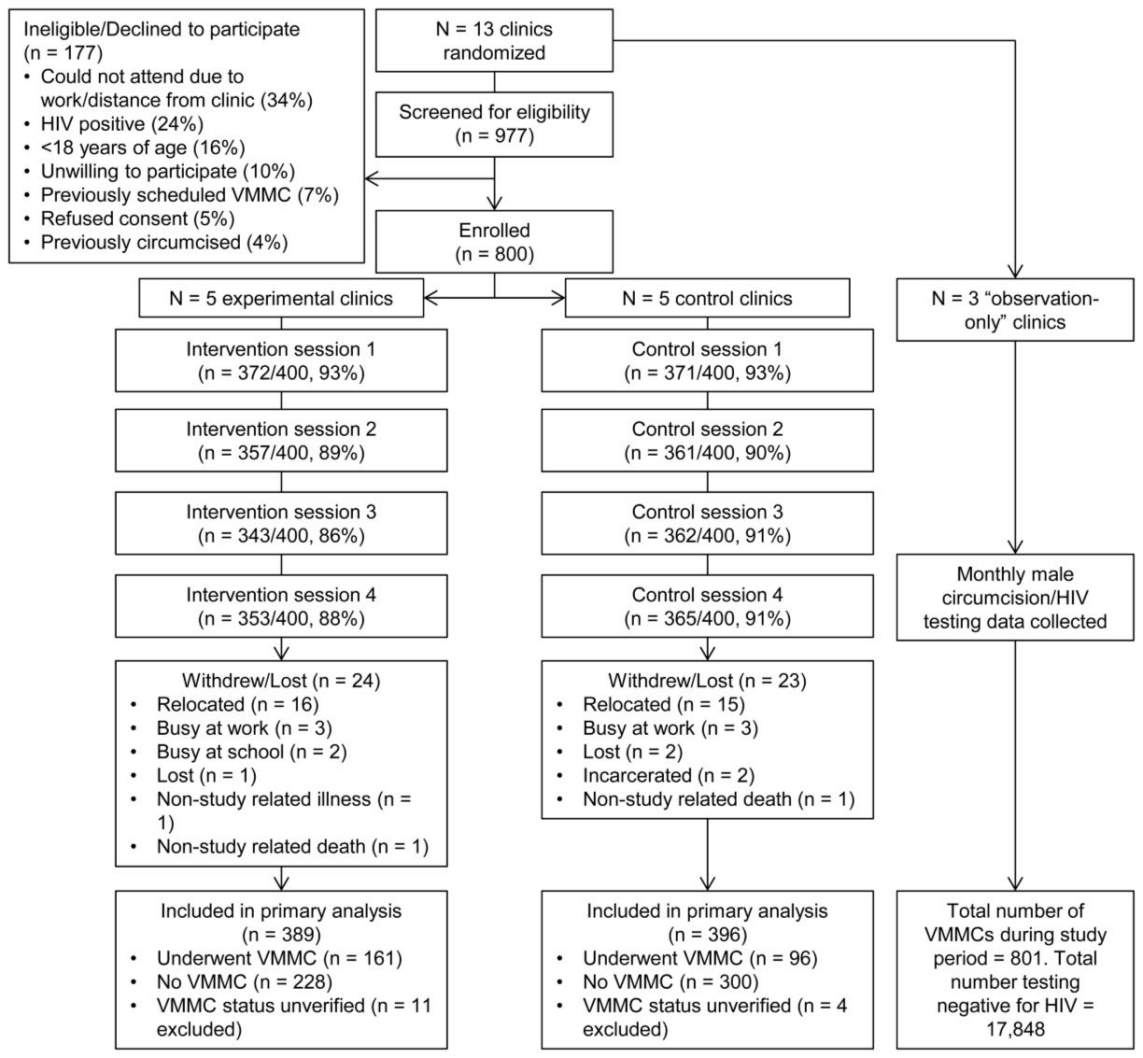
Participant Flow Diagram

Most (82%) participants screened elected to enroll. The most common reasons for non-enrollment were inability to attend study activities due to work/distance from the clinic (34%) or failure to meet study criteria (e.g., 24% HIV-positive, 16% <18 years of age). Loss to follow-up ranged from 0% to 13.8% within clinics, with an average of 5.9%, and was not differential between study conditions (24 Experimental condition lost vs. 23 Control condition, *p* = .8805). Figure 1 presents a study flowchart with further details on participant enrollment, attendance, and analysis. Insert Figure 1 about here

Participants (N = 800) ranged from 18 to 57 years old (mean = 27, sd = 9) with at least 12 years of education (n = 526, 66%). Just under half reported at least part time employment (n = 390, 49%), and half reported an annual income less than 500 Zambian Kwacha (~US$100; n = 423, 53%). Forty three percent (n = 342) were married or cohabitating with a partner and 39% (n = 309) had children. Within conditions, clinics were fairly heterogeneous in demographic characteristics; for example, mean age ranged from 22 to 32 in Experimental clinics and from 25 to 30 in Control clinics. Similarly, the proportion of unemployed participants ranged from 36% to 73% in Experimental clinics and from 39% to 68% in Control clinics. However, no significant differences between study conditions in demographic variables were noted, although there was a trend towards higher unemployment among Control participants (*p* = ·0659; see table 1), and baseline readiness for VMMC did not differ between conditions.

Over the study period, 257 participants underwent VMMC (32%), 528 did not (66%), and 15 participants self-reported VMMC but were unable to be confirmed (2%). Age was associated with VMMC, such that younger participants were more likely to undergo VMMC (*p* = ·0205). Higher levels of education were also associated with increased likelihood of VMMC (*p* = ·0009), as was unemployment (*p* = ·0413). Baseline stage of readiness for VMMC was also related to VMMC (*p* < ·0001). Additional information regarding demographics and VMMC are presented in table 1.

**Figure 2:**
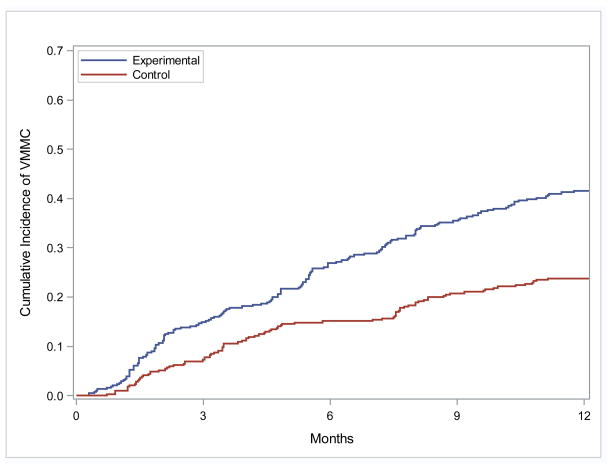
Kaplan-Meier Cumulative Incidence of VMMC

One hundred sixty-one participants in the Experimental condition underwent VMMC (40%), as compared to 96 Control participants (24%). Kaplan-Meier curves were generated to describe the cumulative incidence of VMMC over time (see Figure 2); the 6 month VMMC incidence estimate was 26·9% in the Experimental condition and 15·1% among Controls. Similarly, the 12 month estimate was 41·5% in the Experimental group and 23·7% among Controls.

Prior to completion of the primary analysis, intraclass correlations were computed to describe the similarity within levels of the study. The ICC for participants within clinics was 0·08, and the ICC for participants within cohorts and clinics was 0·23. The results of the primary analysis are presented in table 2. In summary, study condition (Experimental vs. Control) significantly impacted VMMC, such that the adjusted odds of VMMC among Experimental condition participants were 0·69 [95% CI = (0·45, 1·05)] and 0·28 among Controls [95% CI = (0·18, 0·44)]. Thus, participants in the Experimental condition demonstrated approximately 2·45 times the odds of undergoing VMMC as compared to Controls [Adjusted odds ratio (aOR) = 2·45, 95% CI = (1·24, 4·90), *p* = ·0166]. No interactions between condition and covariates were significant. To assess model fit, two R^2^ statistics were calculated; the marginal R^2^ for the model was 0.10, and the conditional R^2^ was 0.26.

Two sensitivity analyses were also performed, using the same methodology as the primary analysis. The first assumed that the 15 participants whose self-reported VMMCs were unable to be verified did indeed undergo VMMC (n = 11 Experimental, n = 4 Control), resulting in 172 VMMCs in the Experimental condition (43%) and 100 among Controls (25%). The results of this analysis were similar to the primary analysis [aOR (Experimental vs. Control) = 2·52, 95% CI = (1·37, 4·64), *p* = ·0081]. The second analysis excluded participants who entered the study already planning to undergo VMMC (n = 99 excluded, n = 58 Experimental, n = 41 Control), resulting in 130 VMMCs in the Experimental condition (39%) and 82 among Controls (23%). The results of this analysis were also similar to the primary analysis [aOR = 2·38, 95% CI = (1·17, 4·86), *p* = ·0228].

**Figure 3:**
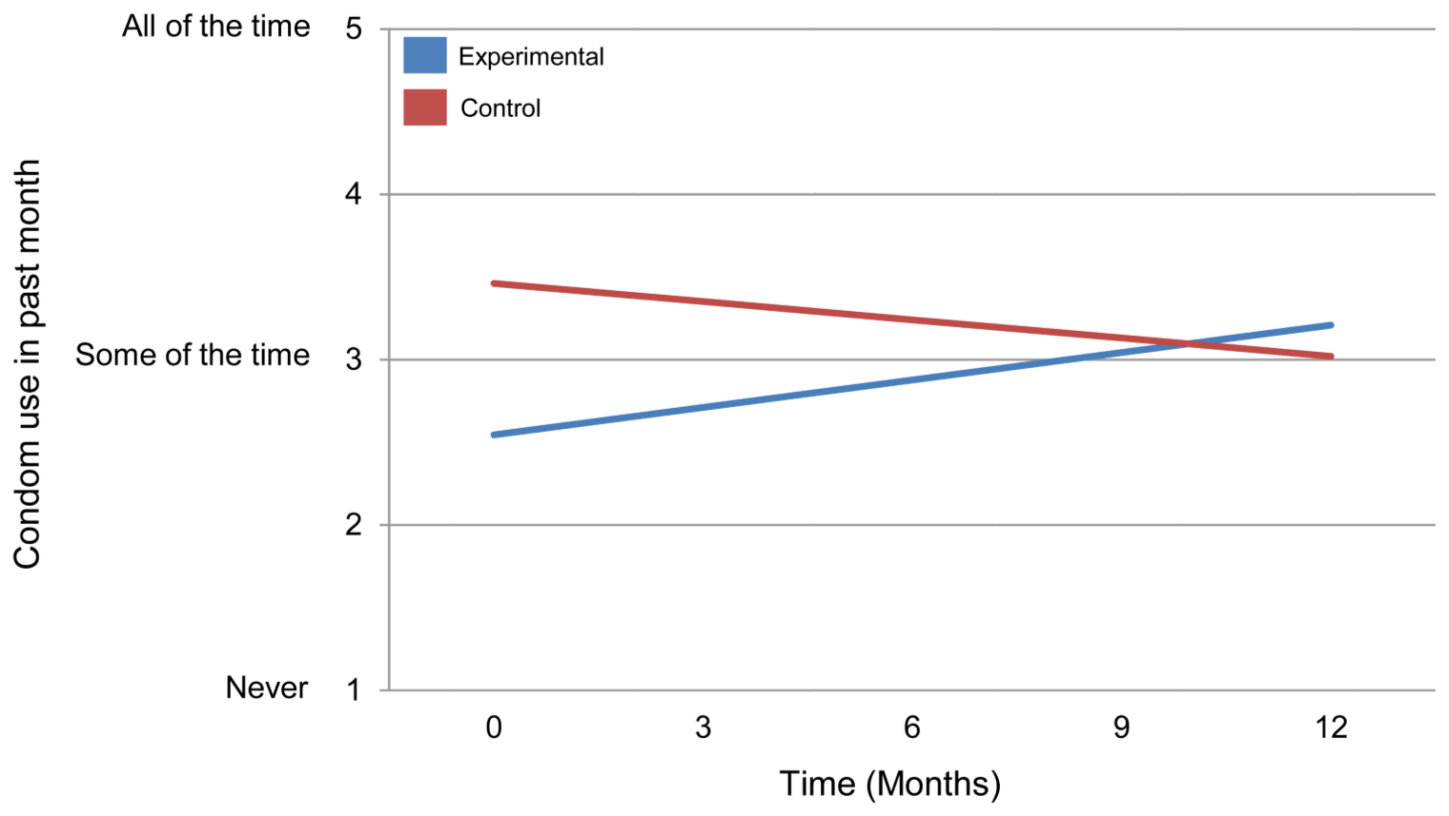
Changes in condom use among participants reporting sex in the month preceding post-VMMC assessment (n = 152)

Participants undergoing VMMC were assessed 3 months after their VMMC. Condom use over time was examined among those participants who reported sexual activity within 1 month of the assessment (i.e., between 2 and 3 months post-VMMC; n = 152; 88 Experimental condition, 64 Control). There was a difference in baseline condom use and a difference in the rate of change in condom use over time between conditions (*p* = ·0155). Baseline condom use was lower among Experimental condition participants relative to Controls (2·54 vs. 3·46; 1 = “Never”, 5 = “All of the time”; *p* = ·0040). However, Experimental condition participants increased their condom use over time, at an estimated rate of ·055 units per month [95% CI = (·01, ·10), *p* = ·0268], whereas condom use among Control participants did not change over time (*p* = ·1979). There was no change in other risk behaviors, e.g., multiple partners, use of alcohol or drugs during sex. Figure 3 shows the changes in condom use graphically.

In addition to study data, the number of men testing negative for HIV and the number undergoing VMMC were collected from all 13 study clinics, each month for 36 months (these data did not include study participants). In total over 36 months, 30,430 men tested negative for HIV and 3,543 underwent VMMC in the 5 Experimental clinics (an 11·64% VMMC proportion, odds of VMMC = 0·13), 42,810 tested negative and 3,392 underwent VMMC in the 5 Control clinics (7·92% VMMC proportion, odds of VMMC = 0·09), and 17,848 tested negative and 801 underwent VMMC in the 3 Observation only clinics (4·49% VMMC proportion, odds of VMMC = 0·05). Using the Observation only clinics as a reference category, the odds ratio for VMMC was 2·80 in the Experimental clinics and 1·83 in the Control clinics (1·53 Experimental vs. Control).

## DISCUSSION

The Spear and Shield intervention significantly increased the number of Zambian men opting to undergo circumcision, with Experimental condition study participants demonstrating approximately 2·5 times the odds of electing to undergo the procedure compared to the Control participants.

Although there was no specific discussion of VMMC in the Control condition, multiple questionnaires at four timepoints during the course of the study assessed their attitudes, feelings, knowledge and intentions undoubtedly produced some interest in Control participants, as illustrated in Figure 2. Exposure to the local media and promotion campaigns may have had a similar effect, causing a modest increase in VMMC, followed by a leveling off at 6 months post-intervention of the number of men undergoing VMMC. In contrast, in addition to a sizable number of participants in the Experimental condition opting for VMMC in the first six months, shown in Figure 2, they continued to opt for VMMC during the six month to one year follow-up. This finding suggests that the intervention may have had a “priming” effect on those who did not elect VMMC in the 6 month post intervention period, such that they might have become more *sensitized* than the Control participants to other environmental influences (e.g., media campaigns, community mobilization, changing social norms) which might have served as a “tipping point” with respect to the decision to undergo the procedure. This comprehensive intervention educated men about multiple HIV prevention strategies, including VMMC, allowing those starting the study in early stages of change time to evaluate their options and make their best choice. These findings support the predictive validity of stages of change for VMMC increasing the likelihood of VMMC uptake over time. These interpretations also lend support to the synergistic potential of combining structured, formal educational, attitude exploration and experiential skill development opportunities such as the Spear and Shield intervention, with less formal and targeted environmental strategies to achieve outcomes that neither could accomplish by themselves.

Virtually all earlier VMMC studies have observed no impact on sexual risk behavior post VMMC. ^6,16^ In contrast, the Experimental condition of the current study demonstrated a significant increase in condom use among those undergoing VMMC. These outcomes support the single previous behavioral intervention study targeting sexual risk behavior post VMMC. ^17^ These studies confirm the added value of nesting VMMC within the context of a comprehensive sexual risk reduction intervention. ^5,18–19^ Consistent with findings from these same studies, neither Control nor Experimental condition participants who underwent VMMC showed changes in numbers of partners or alcohol or drug use before sex. Finally, younger and better educated participants were more likely to undergo VMMC over the course of the study, which appeared to be related to younger persons acknowledging engaging in more high risk behaviors as well as more educated persons being more knowledgeable about the consequences of engaging in such behaviors.

Although not part of the study’s original objectives, it was observed that the number of VMMCs conducted on non-study participants also increased in a “dose-dependent” relationship related to clinic designation, i.e., VMMCs in the Experimental and Control sites increased more than Observation Only sites, and VMMCs in Experimental sites increased more than in Control sites. This “spill-over” effect suggests that the mere presence of VMMC study-related activities at a CHC influence both the clinic and local “culture” in ways previously unrecognized. Future implementation studies should examine the replicability of this observation, and include projections on the public health impact of this phenomenon, if substantiated.

### Panel: Research in context

#### Evidence before the study

A search of studies conducted over the last 5 years designed to increase VMMC acceptability and uptake and prevent behavioral disinhibition was undertaken (Scopus search terms: male circumcision, Africa, intervention, 2009–2014). Search results identified a single post-VMMC behavioral intervention designed to reduce sexual risk behavior that obtained a decrease in sexual risk behavior, e.g., number of partners and unprotected vaginal sex in the experimental condition (Peltzer, 2012), suggesting the short term effect of a brief counseling and HIV-risk reduction session. Several previous studies have found no change in sexual risk behavior post-VMMC (Kibra, 2014; L’Engle, 2014). An additional intervention, providing monetary compensation to increase VMMC uptake, obtained a modest increase in VMMC uptake at higher levels of compensation (Thirumurthy, 2014). However, the current study compensation associated with VMMC was less than half that associated with increasing VMMC rates in the monetary compensation intervention. A recent text messaging intervention did not influence risk behavior (Odeny, 2014), and all other recent studies primarily addressed the acceptability of VMMC and barriers to uptake (Herman-Roloff, 2011; Mugwanya, 2010) but have not addressed interventions to simultaneously increase uptake and decrease sexual risk behaviors.

#### Added value of the study

This study confirms the added value of nesting VMMC within the context of a comprehensive sexual risk reduction intervention. The current study provides the first clinical trial evidence of the potential impact of a comprehensive behavioral intervention combined with increased VMMC availability on the simultaneous increase in VMMC uptake and decrease in sexual risk behavior.

#### Implications of all available evidence

VMMC availability may be “necessary but not sufficient” to encourage men to undergo VMMC. These studies support combining educational and experiential interventions with targeted VMMC promotion strategies to significantly increase VMMC uptake. VMMC is not associated with an increase in sexual risk behaviors; indeed, such comprehensive interventions also may *increase* condom use.

#### Limitations

The primary limitations of the study were a) the relatively small number of clinics in the cluster sample, which might affect external validity, and b) offering only a surgical VMMC option (the uptake of VMMC remains limited by fear of pain, the surgical procedure and adverse events). Novel non-surgical or minimally-surgical techniques are currently being evaluated, e.g., PrePex, ^20^ Shang Ring, ^21^ which may increase acceptability ^22^ when offered as an alternative to surgery. ^23^

#### Future Steps

Most country national plans have focused upon increasing the availability of VMMC services through training of health care providers, in addition to creating community mobilization efforts assisted by intensive media campaigns. However, as highlighted in recent WHO reports, “demand creation” continues to be the major challenge facing those charged with meeting national VMMC goals. Scaling up an evidence-based intervention to simultaneously enhance VMMC uptake as an expansion of post-VCT counseling while training existing CHC health care providers to perform VMMCs may be one of the best ^24^ and most cost-effective ways to significantly impact HIV rates in high incidence countries. ^25,26^

## Supplementary Material



## Figures and Tables

**Table 1:** Demographic characteristics of N = 800 Spear & Shield participants, compared between Experimental and Control conditions and between those who underwent VMMC and those who did not (n = 785 with confirmed VMMC status).

Characteristic	All (N = 800) Mean(sd) n(%)	Experimental group (n = 400)	Control group (n = 400)	t, *p* Chi-square,*p*	Underwent VMMC (n = 257)	Did not undergo VMMC (n = 528)	t, *p* Chi-square,*p*

Age (years)	27(9)	27(9)	27(9)	0·02, .9805	26(8)	28(9)	2·32, ·0205

Employment status				3·38, .0659			4·16, ·0413
Employed	390(49%)	208(52%)	182(46%)		111(43%)	269(51%)	
Unemployed	410(51%)	192(48%)	218(54%)		146(57%)	259(49%)	

Annual Income				0·05, ·8317			0·05, ·8238
≥ 500 ZMK (~$100)	377(47%)	187(47%)	190(48%)		120(47%)	251(48%)	
< 500 ZMK	423(53%)	213(53%)	210(52%)		137(53%)	277(52%)	

Education level				0·20, ·6549			11·07, ·0009
≥ 12 years of education	526(66%)	266(67%)	260(65%)		190(74%)	327(62%)	
< 12 years of education	274(34%)	134(33%)	140(35%)		67(26%)	201(38%)	

Relationship status				1·65, ·1983			3·24, ·0718
Married or cohabitating	342(43%)	180(45%)	162(41%)		97(38%)	235(45%)	
Not married/Not living with partner	458(57%)	220(55%)	238(59%)		160(62%)	293(55%)	

Children				1·52, ·2170			2·40, ·1214
At least one child	309(39%)	163(41%)	146(37%)		88(34%)	211(40%)	
No children	491(61%)	237(59%)	254(63%)		169(66%)	317(60%)	

Desire for (more) children				0·25, ·6177			1·50, ·2202
Yes	349(44%)	178(45%)	171(43%)		103(40%)	236(45%)	
No	451(56%)	222(55%)	229(27%)		154(60%)	292(55%)	

Baseline readiness for VMMC				3·59, ·1662			20·17, <·0001
Precontemplation	431(54%)	207(52%)	224(56%)		112(44%)	312(59%)	
Contemplation	270(34%)	135(34%)	135(34%)		100(39%)	167(32%)	
Preparation	99(12%)	58(15%)	41(10%)		45(17%)	49(9%)	

Note: VMMC = Voluntary Medical Male Circumcision

**Table 2:** Multivariable logistic regression analysis of factors influencing VMMC

Factor	aOR	95% CI (aOR)	*p*

Study condition			
Experimental vs. Control (Ref)	2·451	(1·236, 4·359)	·0166

Readiness for VMMC			
Preparation vs. Precontemplation (Ref)	2·185	(1·266, 3·769)	·0050
Contemplation vs. Precontemplation (Ref)	1·243	(0·836, 1·848)	·2814

Employment status			·0828
Employed vs. Unemployed (Ref)	0·718	(0·494, 1·044)	

Education level			·0026
Higher education vs. Lower education (Ref)	1·829	(1·235, 2·710)	

Age (5 year increase)	0·935	(0·831, 1·053)	·2686

Note: VMMC = Voluntary Medical Male Circumcision; aOR = Adjusted Odds Ratio; Ref = Reference group for odds ratio comparisons

## References

[1] Bailey RC, Moses S, Parker CB (2007). Male circumcision for HIV prevention in young men in Kisumu, Kenya: a randomised controlled trial. Lancet.

[2] Gray RH, Kigozi G, Serwadda D (2007). Male circumcision for HIV prevention in men in Rakai, Uganda: a randomised trial. Lancet.

[3] Mehta SD, Moses S, Agot K (2013). The long term efficacy of medical male circumcision against HIV acquisition. AIDS.

[4] Gray R, Kigozi G, Kong X (2012). The effectiveness of male circumcision for HIV prevention and effects on risk behaviors in a posttrial follow-up study. AIDS.

[5] Lissouba P, Taljaard D, Rech D (2011). Adult male circumcision as an intervention against HIV: an operational study of uptake in a South African community (ANRS 12126). BMC Infect Dis.

[6] Kibira SP, Nansubuga E, Tumwesigye NM (2014). Differences in risky sexual behaviors and HIV prevalence of circumcised and uncircumcised men in Uganda: evidence from a 2011 cross-sectional national survey. Reprod Health.

[7] L'Engle K, Lanham M, Loolpapit M, Oguma I (2014). Understanding partial protection and HIV risk and behavior following voluntary medical male circumcision rollout in Kenya. Health Educ Res.

[8] Njeuhmeli E, Forsythe S, Reed J (2011). Voluntary medical male circumcision: modeling the impact and cost of expanding male circumcision for HIV prevention in eastern and southern Africa. PLoS Med.

[9] Central Statistical Office, M. o. H., National HIV/AIDS/STI/TB Council, University of Zambia, MEASURE Evaluation (2010). Zambia Sexual Behaviour Survey 2009.

[10] Price JE (2014). Behavior change pathways to voluntary medical male circumcision: narrative interviews with circumcision clients in zambia. PLoS One.

[11] Campbell MK, Piaggio G, Elbourne DR, Altman DG (2012). Consort 2010 statement: extension to cluster randomised trials. BMJ.

[12] Donner A, Klar N (2000). Design and Analysis of Cluster Randomization Trials in Health Research.

[13] Health Professions Council of Zambia (2010). Accreditation of Sites for Provision of Male Circumcision Services for HIV Prevention.

[14] Fisher JD, Fisher WA, Misovich SJ, Kimble DL, Malloy TE (1996). Changing AIDS risk behavior: effects of an intervention emphasizing AIDS risk reduction information, motivation, and behavioral skills in a college student population. Health Psychol.

[15] Prochaska JO, Redding CA, Harlow LL, Rossi JS, Velicer WF (1994). The transtheoretical model of change and HIV prevention: A review. Health Educ Q.

[16] Agot KE, Kiarie JN, Nguyen HQ (2007). Male circumcision in Siaya and Bondo Districts, Kenya: prospective cohort study to assess behavioral disinhibition following circumcision. J Acquir Immune Defic Syndr.

[17] Peltzer K, Simbayi L, Banyini M, Kekana Q (2012). HIV risk reduction intervention among medically circumcised young men in South Africa: a randomized controlled trial. Int J Behav Med.

[18] Hayes R, Ayles H, Beyers N, Sabapathy K, Floyd S, Shanaube K (2014). HPTN 071 (PopART): rationale and design of a cluster-randomised trial of the population impact of an HIV combination prevention intervention including universal testing and treatment - a study protocol for a cluster randomised trial. Trials.

[19] Vermund SH, Fidler SJ, Ayles H, Beyers N, Hayes RJ, HPTN 071 Study Team et al (2013). Can combination prevention strategies reduce HIV transmission in generalized epidemic settings in Africa? The HPTN 071 (PopART) study plan in South Africa and Zambia. J Acquir Immune Defic Syndr.

[20] Mutabazi V, Kaplan SA, Rwamasirabo E (2013). One-arm, open-label, prospective, cohort field study to assess the safety and efficacy of the PrePex device for scale-up of nonsurgical circumcision when performed by nurses in resource-limited settings for HIV prevention. J Acquir Immune Defic Syndr.

[21] Kanyago S, Riding DM, Mutakooha E, Lopez de la OA, Siedner MJ (2013). Shang Ring versus forceps-guided adult male circumcision: a randomized, controlled effectiveness study in southwestern Uganda. J Acquir Immune Defic Syndr.

[22] Kigozi G, Musoke R, Watya S (2013). The acceptability and safety of the Shang Ring for adult male circumcision in Rakai, Uganda. J Acquir Immune Defic Syndr.

[23] WHO (2013). WHO Technical Advisory Group on Innovations in Male Circumcision: Evaluation of Two New Adult Devices.

[24] De Cock KM, Jaffe HW, Curran JW (2012). The evolving epidemiology of HIV/AIDS. AIDS.

[25] Martin G, Bollinger L, Pandit-Rajani T, Forsythe S, Stover J (2007). Costing male circumcision in Zambia and implications for the cost-effectiveness of circumcision as an HIV intervention.

[26] Uthman OA, Popoola TA, Uthman MMB, Aremu O (2010). Economic evaluations of adult male circumcision for prevention of heterosexual acquisition of HIV in men in sub-Saharan Africa: a systematic review. PLoS One.

